# Cathodoluminescence excitation spectroscopy: Nanoscale imaging of excitation pathways

**DOI:** 10.1126/sciadv.abq4947

**Published:** 2022-10-07

**Authors:** Nadezda Varkentina, Yves Auad, Steffi Y. Woo, Alberto Zobelli, Laura Bocher, Jean-Denis Blazit, Xiaoyan Li, Marcel Tencé, Kenji Watanabe, Takashi Taniguchi, Odile Stéphan, Mathieu Kociak, Luiz H. G. Tizei

**Affiliations:** ^1^ Université Paris-Saclay, CNRS, Laboratoire de Physique des Solides, Orsay 91405, France.; ^2^ Research Center for Functional Materials, National Institute for Materials Science, 1-1 Namiki, Tsukuba 305-0044, Japan.; ^3^ International Center for Materials Nanoarchitectonics, National Institute for Materials Science, 1-1 Namiki, Tsukuba 305-0044, Japan.

## Abstract

Following optical excitations’ life span from creation to decay into photons is crucial in understanding materials photophysics. Macroscopically, this is studied using optical techniques, such as photoluminescence excitation spectroscopy. However, excitation and emission pathways can vary at nanometer scales, preventing direct access, as no characterization technique has the relevant spatial, spectral, and time resolution. Here, using combined electron spectroscopies, we explore excitations’ creation and decay in two representative optical materials: plasmonic nanoparticles and luminescent two-dimensional layers. The analysis of the energy lost by an exciting electron that is coincident in time with a visible-ultraviolet photon unveils the decay pathways from excitation toward light emission. This is demonstrated for phase-locked (coherent) interactions (localized surface plasmons) and non–phase-locked ones (point defect excited states). The developed cathodoluminescence excitation spectroscopy images energy transfer pathways at the nanometer scale, widening the available toolset to explore nanoscale materials.

## Teaser

Nanosecond temporal electron-based nano-optical coincidence techniques reveals energy transfer pathways

## INTRODUCTION

Light emission spectroscopies reveal materials’ optical excitations. The knowledge of the mechanisms leading from light absorption to emission, i.e., the absorption and decay pathways, is paramount to understanding these excitations’ physics and their applications. Photoluminescence excitation (PLE) spectroscopy is especially suitable for this purpose. In this spectroscopy, the emission intensity is measured as a function of excitation energy. Quantitatively, it directly maps a system’s relative quantum efficiency (QE) as a function of excitation energy. Qualitatively, it permits to access the competition between different relaxation pathways from the selected absorption states toward emission in the selected energy window.

PLE has proven to be invaluable as it provides unparalleled information on the optical properties of materials. Examples include identification of excited exciton states and quantitative measurement of their binding energy in two-dimensional (2D) materials (*1*, *2*), determination of the energy transfer QE in carbon nanotube/porphyrin compounds (*3*), exhaustive characterization of the photophysics of single-photon emitters in nanodiamonds (*4*) and defects in boron nitride (*h*-BN) (*5*), and deep insight into the relaxation pathways in gallium arsenide (GaAs) quantum dots (*6*). The correlative nature of PLE makes it extremely sensitive compared to other absorption techniques. Despite all these advantages and their impact in all fields relying on optical material characterization, from quantum optics to photovoltaics, light diffraction imposes a limit onto spatial resolution for PLE to within a few hundreds of nanometers at best. This severely hinders its application, as the efficiency of the excitation and decay pathways vary drastically at scales much smaller than the wavelength of light (*7*).

Free electron–based microscopies may potentially solve this issue, benefiting from suboptical-wavelength spatial resolution because of the small wavelength of electrons (3.6 pm for 100-keV electrons) and of broadband excitation extending from the infrared to the hard x-ray range (*8*).

To start with, cathodoluminescence (CL) is an emission spectroscopy (*9*–*11*) that measures the light emission spectrum under free electron excitation. The past 20 years have witnessed an impressive success of this technique for nanosciences (*9*, *12*), because it can be seen as a nanoscale equivalent of off-resonance PL for semiconductors (*13*) and of scattering spectroscopy for plasmonic and optical excitations (*14*–*16*). Nevertheless, as the electron excitation is not monochromatic, a CL excitation (CLE) spectroscopy could not be developed solely by mimicking the principles behind PLE.

Attempts to circumvent this problem include the introduction of a novel light intensity autocorrelation method in CL. It indicated that, at least for some excitation pathways, the bulk plasmon creation and decay into multiple electron-hole pairs have to play a role (*17*), as previously proposed (*18*). This technique allows the nanoscale mapping of the energy-integrated relative QE of semiconductor nanowires (*19*, *20*), without resolving the absorption energy at the origin of the luminescence, therefore failing to resolve the exact physical origin of the absorption and decay pathways. Nevertheless, the plethora of other possible pathways to emission has neither been investigated nor considered.

To solve this problem, this absorption information can, in principle, be retrieved with a companion relativistic electron spectroscopy, the electron energy-loss spectroscopy (EELS). It can be described as a nanometer-scale counterpart of absorption (or more precisely extinction) spectroscopy (*16*). It has been used in combination with CL (*16*, *21*, *22*) to gain insights into the physics behind light emission upon electron scattering. The excitation energy leading to each CL event is encoded in the individual electrons constituting an EELS spectrum. Unfortunately, this information is lost with the current time-averaged technologies, making impossible the investigation of excitation-to-emission pathways at the nanometer scale.

Here, we demonstrate CLE with nanometer-scale spatial resolution over a broad energy range (from the visible to the soft x-ray, 2 to 620 eV) in a scanning transmission electron microscope (STEM). Our approach relies on a specially developed coincidence scheme between inelastic electron scattering and photon emission events. If the temporal information of both these events is known, correlation can be performed to unveil the probability of each of the energy transfer pathways. CLE spectra are constructed with EELS events that are time correlated with a photon emission, while energy relative QE spectra are given by the ratio of CLE and total EELS spectra. As a proof of principle of CLE, we focused on representatives of the two main families of optically relevant materials, plasmonic nanoparticles for the photonic materials and defects in semiconducting materials for the luminescent ones. Studying CLE on Au nanospheres embedded in SiO_2_, two light emission pathways are identified: surface plasmons (SPs) for the Au and transition radiation (TR) for the SiO_2_ and Au. The direct energy and time correlation between absorption and emission for these excitations, known to be phase locked (coherent) with the exciting electron, is a confirmation of the relevance of CLE. With CLE on *h*-BN flakes, TR was also detected. This signal is usually in the background of EELS spectra, evidencing the extremely high sensitivity of the technique, with a typical improvement of two orders of magnitude. CLE was used to explore the decay pathways leading to the excitation and emission of the 4.1-eV defect in *h*-BN. All excitations, from the near-band edge (NBE) to the core losses, including the bulk plasmon, are demonstrated to participate in photon emission. The bulk plasmon is experimentally confirmed as the main absorption pathway. Nevertheless, the relative QE first peaks at the NBE energy and is followed by a linear increase up to the maximum energy in the soft x-ray energy range (620 eV), which has not yet been observed. The NBE pathway is unexpectedly the most efficient excitation channel for defect light emission, up to absorption energies of 15 eV. Last, spatially resolved CLE in *h*-BN reveals the spatial variation of the excitation and decay pathways with a 125-nm spatial resolution. STEM-CLE, on that account, has proven to be a nanometer-scale counterpart of PLE.

## RESULTS

**Fig. 1.**
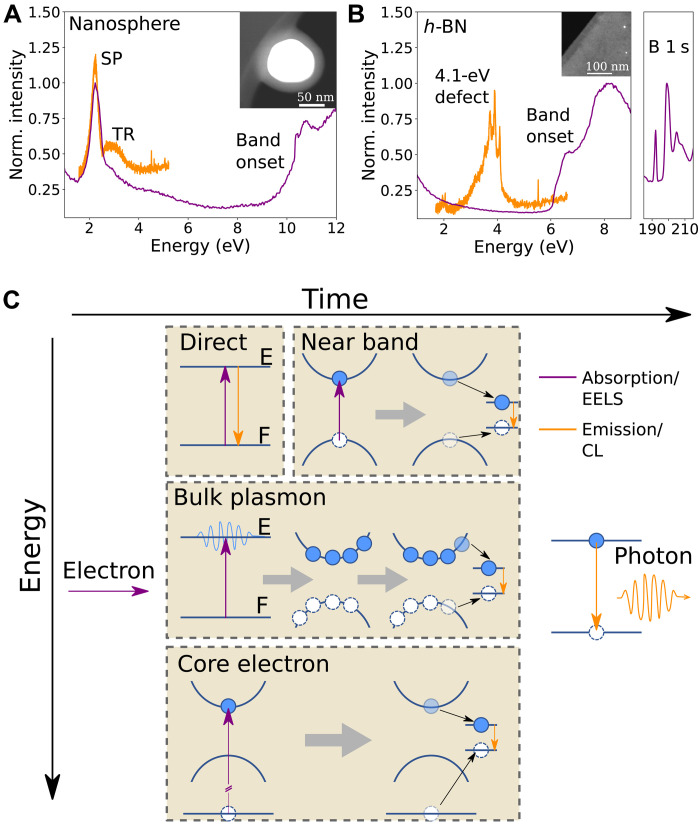
Photon emission pathways upon electron scattering. (**A** and **B**) Time-averaged CL (orange) and EELS (purple) spectra of an Au/SiO_2_ nanosphere and a thin *h*-BN flake show different absorption and emission features, described in the text. From these correlative time-averaged spectra, one cannot identify which absorption transitions lead to light emission. The small intensity emission at ≈2 eV in the *h*-BN CL spectrum is a replica of the 4.1-eV defect emission due to the diffraction grating. The insets show images of the nanosphere and the *h*-BN edge. CL and EELS spectra have been normalized and shifted vertically for clarity. (**C**) A relativistic inelastic electron scattering event in a solid can generate different excitations (vertical purple arrows): direct optical transition, NBE transition, bulk plasmon excitation, and core-level transitions. Excitations not involving single particles (excitons, bulk and SPs, etc.) are represented between a fundamental (F) and excited (E) state. These can relax through different pathways, leading to the excitation of a final optically bright energy level and to photon emission (vertical orange arrows).

In the following, we concentrate on Au nanospheres embedded in SiO_2_, mainly showing SP resonances at around 2.2 eV both in absorption and emission, and *h*-BN flakes, with a dominating NBE and plasmonic absorption and strong 4.1-eV defect emission (*23*, *24*), as clearly seen on the absorption (EELS) and emission (CL) spectra in Fig. 1 (A and B). Nevertheless, because of their time-averaged nature, these spectra alone cannot reveal the excitation-to-emission pathways shown schematically in Fig. 1C.

Electron scattering in matter leads to light emission through different processes, extending in wide energy (from millielectron to kilo–electron volt) and time (from femtosecond to microsecond) ranges. In Fig. 1C, optical transitions are represented by vertical arrows and relaxation pathways by black arrows, with qualitative temporal axis from left to right.

To understand CLE, it is necessary to know how EELS and CL spectra relate to the processes described in Fig. 1C. Every inelastically scattered electron must undergo an initial excitation (purple arrow in Fig. 1C) that can be measured with EELS. This encompasses TR, NBE excitations, core-level excitations (*8*), bulk (*25*) and surface (*26*) plasmon excitations, phonon excitations (*27*, *28*), and exciton excitations (*22*). TR occurs when a relativistic electron crosses a dielectric discontinuity and is often missed in the presence of other excitations in the same energy range, because of its small oscillator strength (Fig. 1, A and B). The NBE of semiconductors is easily detected in EELS (Fig. 1, A and B, for SiO_2_ and *h*-BN), especially with modern electron monochromator technologies (*28*). Core-electron spectroscopy is widely used for chemical mapping and allotrope identification (*8*) down to the atomic scale (*29*).

After having been created through the above-detailed absorption process revealed by EELS, these distinct excitations over a wide range of energies can lead to photon emission, detected with CL in the infrared-ultraviolet range, through different relaxation pathways, some of which are still not understood. TR and SP are typical of photonic materials, characterized by a phase-locked emitted photon relative to the exciting electrons (*15*). As a consequence, extinction (EELS) and emission (CL) spectra are similar, with only slight shifts expected (*16*), and therefore, we expect a CLE spectrum to closely resemble a CL spectrum. In luminescent materials, absorption and decay pathways are expected to be more complex upon electron excitation. As depicted in Fig. 1C, NBE, bulk plasmons, core-level excitations, or direct excitations can lead to the emission of light, and we expect the CLE to be quite different from the EELS. A microscopic description of the weight of each of the energy transfer processes is still not available.

**Fig. 2.**
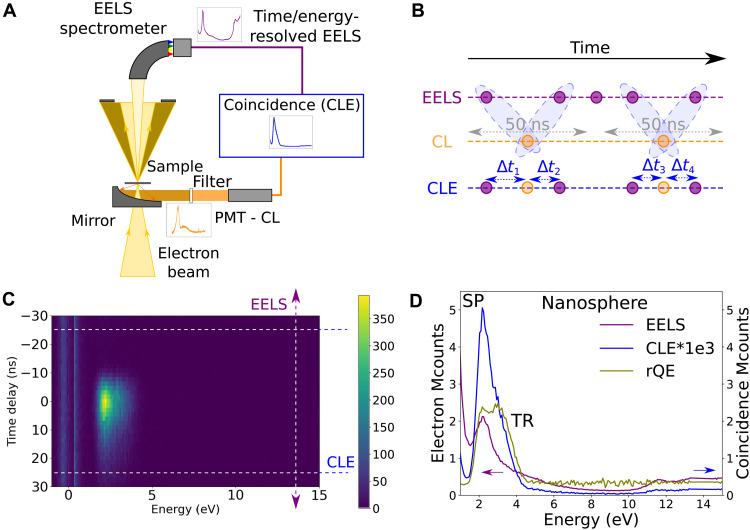
CLE in a STEM. (**A**) Sketch of the experimental setup: A 60- to 100-keV electron beam is focused in a nanometer spot that can be scanned along the surface of a sample. Time-resolved CL events (orange) are collected through a parabolic mirror and detected, after passing through a filter, with a PMT, and time-resolved EELS events are measured by a TimpePix3 detector after an EELS spectrometer. These are stored in an ordered list, used to produce coincidence spectra (blue). (**B**) A search algorithm is used to find electrons that are within ±25 ns of a detected photon, from which a 2D histogram of time delay versus electron energy loss is reconstructed. (**C**) This 2D histogram shows the temporal evolution of the loss spectrum a as function of delay to a detected photon. (**D**) With this information, total EELS (all detected electrons), the CLE (±5 ns from Δ*t* = 0 ns), and the relative QE (rQE) for an Au/SiO_2_ nanosphere were calculated. For the nanosphere, the SP and TR decay channels are efficient photon emission pathways. TR is less visible in the average EELS spectrum, and it is exacerbated in the CLE.

An emission (CL) event is necessarily preceded by an absorption or extinction event at a given energy (EELS). This relation is temporal in nature and is lost in commonly time-averaged EELS spectra where all potential EELS events corresponding to the same emission are summed. It is, however, stored in the probability of each electron scattering event and photon emission. This can be retrieved by generating coincidence histograms of electron energy-loss and photon emission events (described in what follows; in Fig. 2, A and B; and in the Supplementary Materials). Coincidence electron spectroscopy and microscopy have been performed in the past, for example, coincidence of EELS with secondary electron or x-ray emission (*30*–*32*). EELS-CL coincidence has been performed for discrete selected EELS energy ranges (*33*, *34*), but the relative QE as a function of energy and its spatial dependence has not been measured.

To achieve CLE, a temporal resolution below the time interval between events, given by the electron current (typically 1 electron every 16 ns for 10 pA), is required for all energy-loss events of interest. With this in mind, we implemented an EELS-CL setup in a STEM, displayed in Fig. 2A. In Fig. 2, we illustrate the principle of CLE on the simplest case of the plasmonic particles. For EELS, a Timepix3 detector was used (*35*). The detector provides sub–10-ns time resolution over arbitrary energy ranges determined by the resolution power of the electron spectrometer and the Timepix3 pixel size. In addition, the particular detector used (CheeTah, from Amsterdam Scientific Instruments) has two time-to-digital converters (TDCs), allowing to append timestamps from external signals into the original electron data flow. Photon emission events were detected with a photomultiplier tube (PMT) working in the 2.0- to 5.0-eV energy range. The PMT output is directly connected to one of the Timepix3 TDC lines. Electron and photon arrival times were stored in a list, along with the electron energy loss. The response time of the detection scheme is ≈5 to 25 ns. We used a search algorithm [see methods in the Supplementary Materials and the code available at Zenodo (*36*)] to find electrons that are within ±25 ns of a detected photon, from which a 2D histogram of time delay versus electron energy loss is reconstructed (Fig. 2C). This 2D histogram shows the temporal evolution of the loss spectrum as a function of delay to a detected photon.

From these, we reconstructed a 2D histogram of electron energy-loss events as a function of time delay to a photon emission (Fig. 2C). Because of the typical lifetimes of the CL events (SP and TR in subpicoseconds and defect emission in subnanoseconds), the CLE spectrum is extracted from the shortest time delays given the time response of the experiment (±5 ns), within which coincidence above the long delay limit was observed. For longer lifetimes, larger time integration should be considered. The CLE spectrum resembles an EELS spectrum but weighted by the photon emission probability (Fig. 2D). Last, the ratio of the CLE and the noncoincidence EELS therefore provides the relative QE of different absorption processes (Fig. 2D). It highlights differences between competitive radiative and nonradiative pathways.

For the nanospheres (Fig. 2D and figs. S2 and S3) with the electron beam incident on the SiO_2_ shell, photon emission is due to the Au nanosphere SP decay (2.0 to 2.4 eV) and SiO_2_ TR (2.6 to 4.0 eV), while higher energy losses do not contribute to light emission in the emission detection range. This is a reassuring observation, as photonic modes such as plasmons or TR are created in phase with the field of the electron and can only be created by loss events with energies in the same range as the emission ones (*15*). In the same line, the relative QE is featureless above the SP and TR energies. This is expected as, for a phase-locked excitation, we do indeed expect all the light emitted at a given frequency to have been triggered by an extinction event at the same energy; no energy is transferred from higher frequencies. A similar observation is reported by Feist *et al.* (*37*) on micrometric photonic structures using an equivalent EELS-CL coincidence experiment. We note that, in general, some spurious coincidences (section S7) are detected, but this cannot be avoided: Part of them stems from detector noise (PMT photocathode and ambient light leakage) or from the poissonian nature of the electron source used (this could be improved with a pulsed electron source or a better detector temporal point spread function). Also, the SP and the TR peaks observed that are modulated by the PMT response to photons are a function of wavelength: Coincidence events outside the PMT response range are missed.

**Fig. 3.**
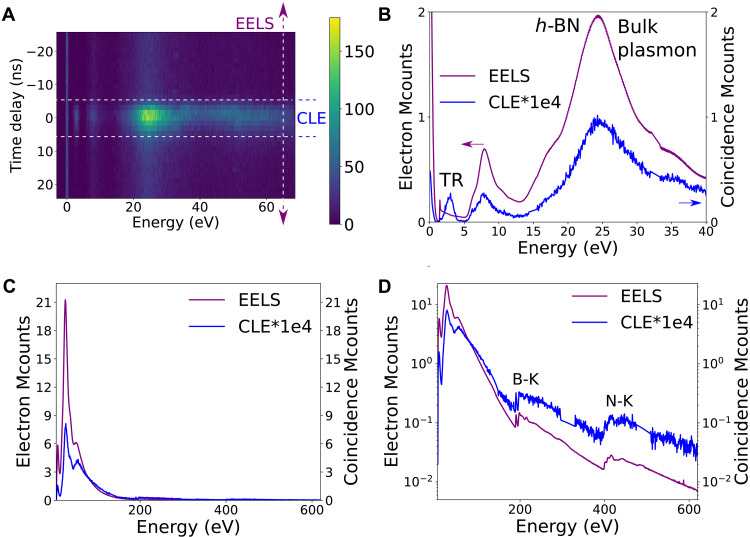
CLE of *h*-BN. (**A**) 2D coincidence histogram for a thin *h*-BN flake. (**B**) EELS and CLE spectra of a *h*-BN thin flake. (**C** and **D**) EELS and CL of a different *h*-BN flake up to core losses in linear and logarithmic scales, respectively. In these spectra, TR, NBE, and all energies above it contribute to photon emission, even up to 600 eV. Detector junctions appear at around 15 and 33 eV (A) and around 150, 320, and 500 eV and are interpolated in spectra (D).

As the observation of these coincidence events and decay channels in the simplest case of phase-locked excitations validates our methodology, we turn to the more involved case of semiconductor emission. In a thin *h*-BN flake (<50 nm), the CLE spectra show contributions from TR, NBE, bulk plasmons, and higher energies toward light emission (4.1-eV defect and TR, included in our detection range). As discussed, the contribution from TR is usually missed in EELS spectra because of its small cross section. As a matter of fact, they are invisible in the EELS and CL spectra of Fig. 1B. In the CLE spectra (Fig. 3, A and B), their contribution (at 1 × 10^−5^ event counts compared to the regular EELS counts, a typical two orders of magnitude better sensitivity than previously demonstrated) is revealed, as a signature of the high sensitivity of CLE, much along the lines of PLE. The emission of the 4.1-eV defect is peaked between 3.65 and 4.1 eV, while that of TR is much broader. The use of a broadband filter (3.65 to 4.1 eV) filters out part of the TR contribution (fig. S5).

The contribution from the defect emission can be seen in the NBE, bulk plasmon (Fig. 3B), and core losses up to the maximum detected energy (Fig. 3 and fig. S4). From the CLE, we prove experimentally the common assumption that the bulk plasmon (24.4 eV) is indeed the main source of electron-hole pairs that, after relaxation, leads to the emission of the 4.1-eV defect. Nevertheless, the NBE absorption is demonstrated to be a non-negligible source of emission and core losses to be also a possible excitation path.

**Fig. 4.**
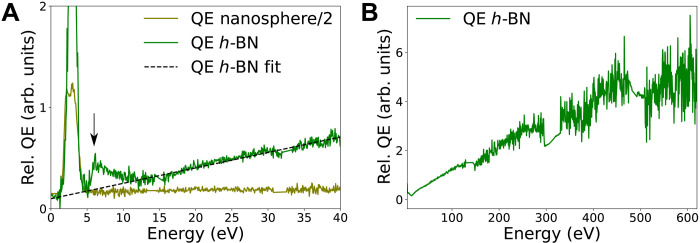
Relative QE. (**A**) Relative QE of a nanosphere (Fig. 2) and a thin *h*-BN flake (Fig. 3). NBE losses are more efficient pathways for light emission than energies below the bulk plasmon. Above 15 eV, the relative QE increases linearly. The nanosphere relative QE was divided by 2 for clarity, which is shown in detail in fig. S2. (**B**) Thin *h*-BN relative QE up to core losses, showing the B and N K-edges. Detector junctions appear at around 15 and 33 eV (A) and around 150, 320, and 500 eV and are interpolated in spectra (B).

The investigation of energy-resolved relative QE (Fig. 4) permits to better understand the physics of energy transfer from absorption to emission in semiconductors. First, we see that contrary to phase-locked excitations, for which the high energy relative QE is completely zero, that related to the 4.1-eV defect has a nonzero and nonmonotonic behavior. Second, NBE is notably a more efficient excitation channel for the emission of the 4.1-eV defect than other excitations up to absorption energies of 15 eV. Above this energy, the efficiency for photon emission increases linearly, up to the maximum energy we have measured (620 eV), i.e., over an energy range much larger than achievable with PLE. The extrapolated linear trend at low energy crosses zero at the bandgap energy. This can be tentatively explained as follows. Each excitation at energy loss *E* can lead to the generation of at most *N* electron-hole pairs and then at most *N* photons, where *N* = *E*/*E_g_* and *E_g_* is the bandgap energy. Below the bandgap energy, the number of electron-hole pairs generated is zero. The optical bandgap of *h*-BN measured using EELS is around 6.0 eV (*38*, *39*). Assuming that the last step to the 4.1-eV defect emission is the NBE electron-hole pairs, the linear trend is deduced. With this, the peak in the relative QE at the NBE energy is reminiscent of an unforeseen resonant effect that will require further theoretical investigation. Direct resonant excitation of defect states by fast electrons has yet to be observed.

Now that the principle of the CLE is established, we turn to the possibility of mapping the different pathways directly in real space. The proposed spectroscopy scheme allows for coincidence mapping, of which more details are reported elsewhere (*35*). The 4.1-eV emission in *h*-BN (Fig. 1C) is known to arise from single point defects (*24*). For each single defect, the CL excitation area forms an intensity spot of ≈80 × 80 nm^2^ wide. We performed CLE mapping by rastering a nanometer-sized beam on the sample and collecting a full CLE spectrum corresponding to emission in the 3.65- to 4.13-eV range at each pixel of the scan. From this, CLE maps can be created by filtering over different absorption (EELS) ranges.

**Fig. 5.**
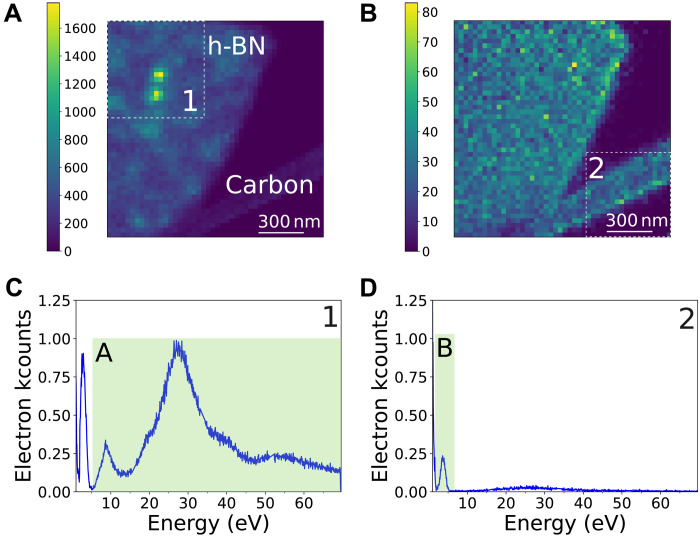
Spatially resolved CLE maps in *h*-BN. (**A**) CLE energy-filtered map above 6.5 eV, the NBE energy, showing multiple localized absorption maxima that lead to the emission of the 4.1-eV defect. (**B**) CLE energy-filtered map between 2 and 5 eV, showing where TR occurs. Both the *h*-BN thin flake and the amorphous carbon support (bottom right, which is the support for *h*-BN in the TEM sample) show absorption leading to photon emission. (**C** and **D**) CLE spectra of regions marked 1 and 2 in (A) and (B), with marked integrated ranges for maps A and B, respectively.

These time-resolved maps permit disentangling the different decay pathways in space and energy, with a 32-nm spatial sampling. The two bright features in the image are separated by 125 nm. The CLE map filtered above 6.5-eV energy loss shows two sharply localized intensity spots consistent with the observation of 4.1-eV localized defects (Fig. 5A). On the contrary, the CLE map filtered between 2 and 5 eV (Fig. 5B), on the peak linked to TR, shows that both the *h*-BN flake and the thin amorphous carbon support (of the TEM grid; see methods in the Supplementary Materials) exhibit coincidence events distributed in a relatively uniform manner. We note that we could not identify any specific absorption signature of the defects at their absorption energy. Coincidence measurements with better EELS spectral resolution might reveal it in the future. Also, the spatial resolution is essentially dependent on that of the CL, which is limited by the diffusion lengths in the materials. One can expect a few nanometers of spatial resolution in other materials, such as III-N heterostructures (*12*).

## DISCUSSION

In conclusion, we demonstrated spatially resolved CLE, which encompasses the main advantages of PLE (high-sensitivity measurement of the relative QE and consequent insight between multiple light emission decay pathways) with that of electron spectroscopies (wide energy range and nanometer-scale spatial resolution). Numerous applications of CLE are expected for nanomaterials, ranging from the optimization of single-photon sources (*4*), the unveiling of the role of nanometer- to atomic-scale features on the optical properties of transition metal dichalcogenide monolayers by mapping the excitons’ binding energy (*1*, *2*), to the characterization of previously unknown optical materials such as hybrid perovskites (*40*) and others yet to be found and understood. The spectromicroscopy scheme described requires only time-resolved electron and photon detectors, being implementable in any electron microscope. Therefore, it is applicable to any object compatible with STEM observation, should they be photonic (plasmonics systems, photonic bandgap materials and waveguides) or luminescent (quantum wells, quantum dots, and single-photon emitters) (*9*, *12*). The current applications of the setup in the time domain are limited by the electron detector temporal resolution. Improvements in the near future are expected with the new Timepix4 detector (*41*), with fast deflectors or with the use of pulsed electron guns (*42*–*44*). Photon and electron energy-resolved experiments in the core-level range with better temporal resolution should give further hints on the microscopic physics behind the relaxation pathways. In addition, as the number of emitted photons per electron per energy is lost using single-pixel detectors, the use of multiple PMTs or 2D arrays of detectors solves this, giving access to excitation energy–resolved Hanbury Brown and Twiss interferometry (*45*) for energy-resolved retrieval of quantum statistics, energy efficiency for total photon yield, and excited energy-resolved bunching experiments (*17*). As for PLE, this technique resolved in emission and absorption energy will allow one to assign specific energy bands to each observed transition but now with nanoscale spatial resolution. Last, polarization-dependent EELS (*46*, *47*) and CL will give us an almost ideal nano-optics to probe excitation symmetries.

## METHODS

Coincidence EELS-CL experiments were performed on a modified Vacuum Generator (VG) HB501 STEM equipped with a cold field-emission source, an Attolight Mönch light collection system, a liquid nitrogen–cooled sample stage, and a Cheetah Timepix3 (manufactured by Amsterdam Scientific Instruments) event-based direct electron detector. More details about the experimental setup and event-based detection can be found in the work of Auad *et al.* (*35*). Beam current in coincidence measurements was typically from 1 to 10 pA, and convergence half-angle of 7.5 mrad was used.

High-energy resolution EELS and CL measurements and high-angle annular dark-field imaging (Fig. 1, A and B) were performed on monochromated and Cs-corrected ChromaTEM modified Hermes200 STEM from NION. Spatially resolved data are acquired by scanning a subnanometer electron beam on the sample. Beam current in the order of 200 pA and convergence half-angle of 25 mrad were used for the experiments. EELS dispersion was set to either 25 meV per channel for low losses (Fig. 1, A and B) or 270 meV per channel for core losses (Fig. 1B). CL used a Mönch system from Attolight, fitted with a diffraction grating giving a wavelength resolution of 0.34 nm (about 2 meV at 500 nm in wavelength).

Experiments were performed with 60- and 100-keV electron kinetic energy. *h*-BN flakes suffered damage at 100 keV on experiments on the VG microscope, where the sample chamber vacuum conditions are degraded (higher pressure and water partial pressure) in comparison to the ChromaTEM microscope. Data acquisition was handled with Nion Swift 1.5 (ChromaTEM) and 1.6 (VG) python-based microscopy control application.

Two samples were used for experiments. The first consists of gold silica core-shell nanospheres from nanoComposix. Their total diameter measured by TEM is 140 ± 10 nm (100-nm core with a 20-nm shell) as stated by the manufacturer. The nanosphere solution was further diluted in spectral-quality ethanol in proportion of 1:2. One drop of the final solution was then drop-casted on a conductive lacey carbon film (tens of nanometers thick) supported on a copper TEM grid (Agar Scientific), and the extra volume was absorbed by filter paper (Whatman).

The second sample is made of *h*-BN flakes (*23*). Thin *h*-BN flakes were prepared by liquid-phase exfoliation from *h*-BN monocrystals dispersed in 1 ml of spectral-quality isopropanol (Carlos Erba) and then sonicated for 15 min. Three drops of the solution were then successively drop-casted onto the TEM grids (suspended by tweezers) containing the Au/SiO_2_ nanospheres. The grid was left to dry until total evaporation of the solvent. This *h*-BN flakes with thickness below 50 nm were chosen to ensure that fast electrons suffer statistically at most one scattering event on the sample (*8*) (sample relative thickness below one mean free path for inelastic scattering).
