# Supplementary material for: Cathodoluminescence excitation spectroscopy: Nanoscale imaging of excitation pathways
Source: Sci Adv. 2022 Oct 7;8(40):eabq4947. doi: 10.1126/sciadv.abq4947 (PMC9544325; doi:10.1126/sciadv.abq4947)
Supplement: Supplementary file 1 — Supplementary Text Figs. S1 to S5 [file sciadv.abq4947_sm.pdf]

Science Advances
AAAS

Supplementary Materials for

Cathodoluminescence excitation spectroscopy: Nanoscale imaging of excitation pathways

Nadezda Varkentina *et al.*

Corresponding author: Luiz H. G. Tizei, luiz.galvao-tizei@universite-paris-saclay.fr;
Mathieu Kociak, mathieu.kociak@universite-paris-saclay.fr

Sci. Adv. **8**, eabq4947 (2022)
DOI: 10.1126/sciadv.abq4947

This PDF file includes:

Supplementary Text
Figs. S1 to S5

Supplementary Text

S1. Description of coincidence seeking algorithm

Before applying the coincidence-seeking algorithm, electron events in Timepix3 were cluster-corrected following a procedure that can be found in ref. (35). The time of arrival of each electron event is compared with the time of arrival list from the photon events (after both being sorted by time). A coincident electron is found when its time lies within a given time interval (±25 ns or ±50 in this work) centered at the first-matching photon time. Instead of pairing every electron with all the elements of the photon list, a sliding window algorithm is performed, which allows to increase performance by as much as a factor of 10 due to the effective photon list's reduced size. The algorithm standard output consists of a list of energy-loss indices along their associated time delay. For the hyperspectral dataset reconstruction (Fig 5), supplementary events from the microscope scanning unit is used. This procedure can be found in detail in ref. (35). Time delay (Δt) was set to zero at the maximum of coincidence counts. Its absolute value is meaningless as it includes electronic and propagation delays.

S2. Projections of the 2D histogram of Fig. 3A

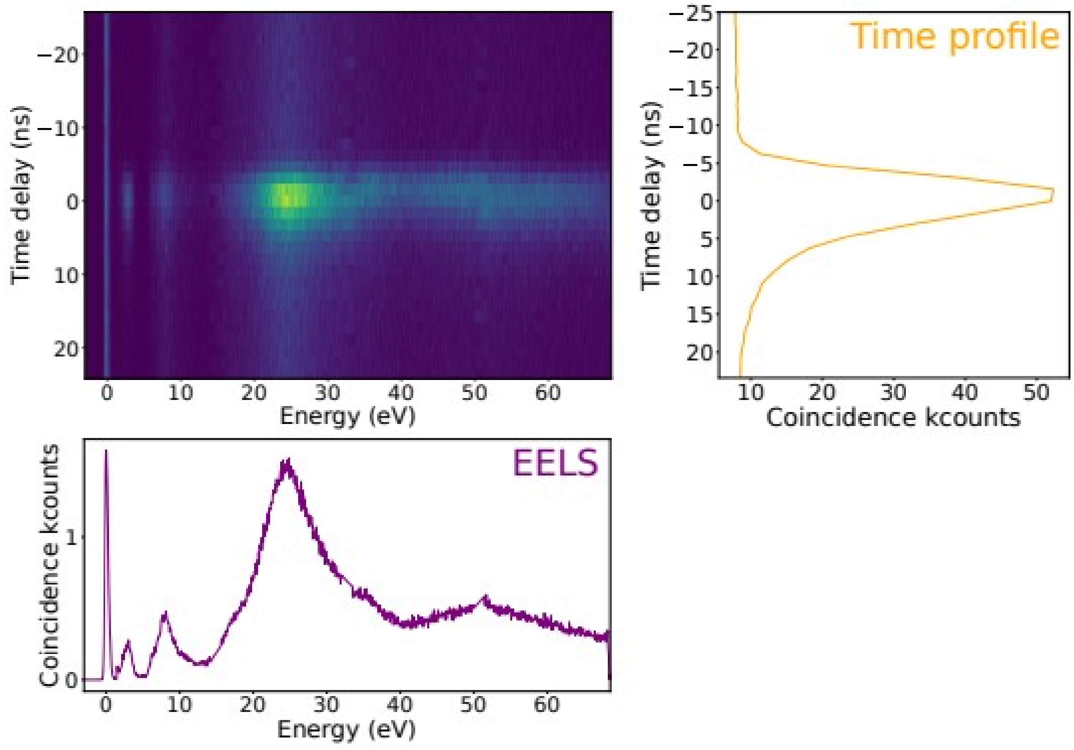

Fig. S1.Projections along the energy and time delay axes of the 2D histogram of Fig.3A: the 2D time delay/energy histograms, as the one of Fig. 3A, depict the probability of a specific energy loss to lead to a photon emission after a given time delay. To facilitate the visualization, projections along the energy (right) and the time delay (bottom) axes are shown.

S3. EELS, CLE, relative QE of nanosphere in Fig. 2 and 4A

A new graph with the same spectra as in Fig. 2B and 4A is presented in Fig. S2, to show in detail the double peaks due to SP and TR in the relative quantum efficiency of a Au/SiO_2 nanosphere.

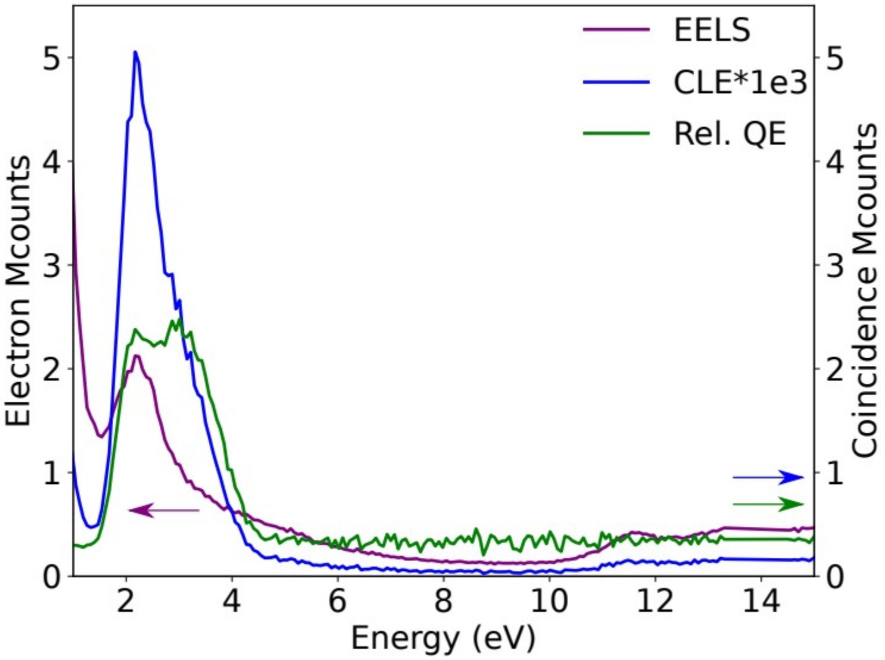

Fig. S2. EELS, CLE and relative QE of the nanosphere in Fig. 2A and 4A: The three spectra were plotted in a narrower energy band and in the same graph to show the double peak structure in the relative quantum efficiency (green). The first peak appears due to the SP resonance, seen in the EELS spectrum, while the second occurs due to TR. Its presence is already indicated in the CLE spectrum (blue), by a shoulder on the SP resonance.

S4. CLE nanosphere in impact and aloof geometry

EELS, CLE and relative quantum efficiency for a nanosphere in the aloof (electron beam outside the SiO2 surface) and impact (electron beam on the SiO_2 layer) geometries.

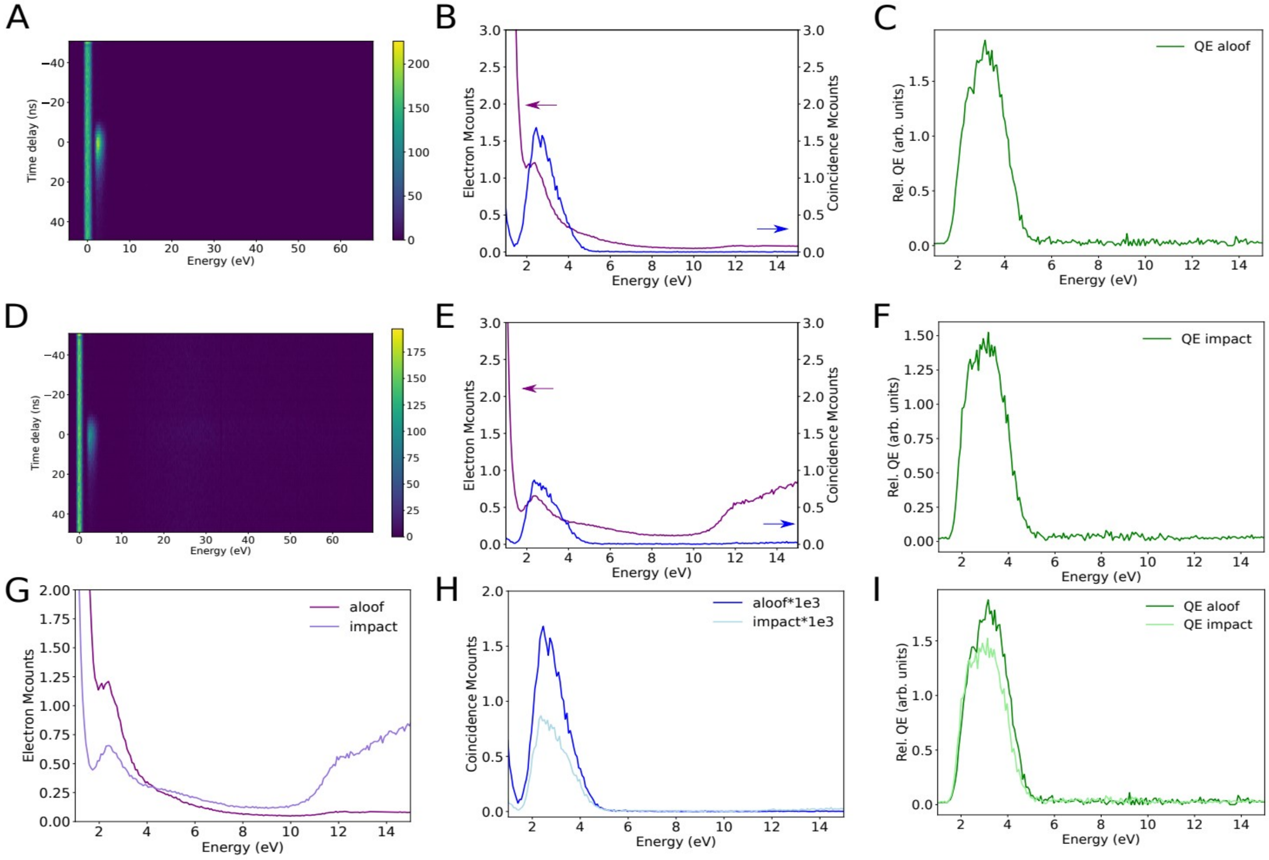

Fig. S3. EELS, CLE and relative QE of a nanosphere in aloof and impact geometries: (**A-C**) EELS, CLE and relative QE for an Au/SiO_2 nanosphere in aloof geometry. (**D-F**) EELS, CLE and relative QE in impact geometry. (**G-I**) Comparison of EELS, CLE and relative QE between aloof and impact geometries. Type or paste caption here. Create a page break and paste in the Figure above the caption.

S5. CLE of h-BN up to core losses

The relative QE of h-BN for the 4.1 eV emission has a linear dependence up to 620 eV shown in Fig. S4, within the currently achievable signal-to-noise ratio. B and N K-edge fine structure is visible in the CLE spectra. No spectral fine structure appears in the relative QE, but the noise level is still high. The minimum of relative QE is visible at around 15 eV, as well as the decrease in relative QE between 6.5 and 15 eV (pointed by green vertical arrows in S4E-F). Panels A-C are the same as those in Figs. 3C-D and 4A for comparison.

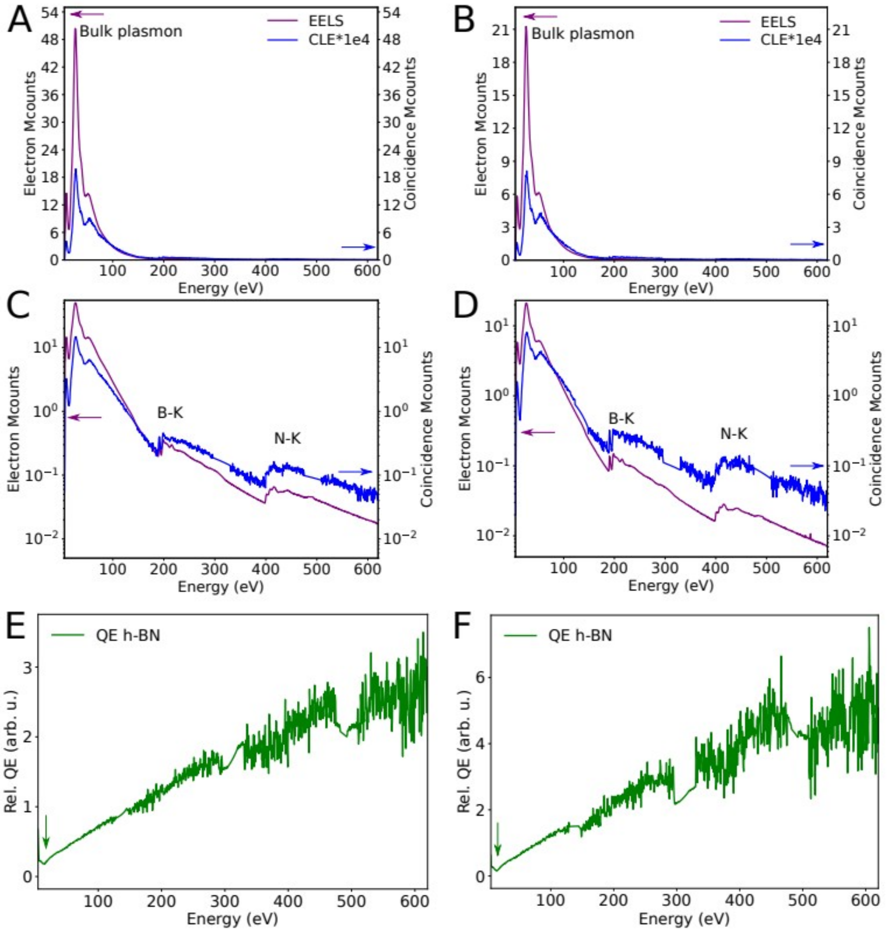

Fig. S4. EELS, CLE and relative QE of a nanosphere in aloof and impact geometries: (**A-B**) EELS and CLE of two h-BN flakes measured up to 620 eV. (**C-D**) Same spectra as in A-B but with logarithmic scales. B-K and N-K mark the boron and nitrogen EELS K edges. (**E-F**) Relative QE for the two measured flakes. The minimum of relative QE is visible at around 15 eV, as well as the decrease in relative QE between 6.5 and 15 eV (pointed by green vertical arrows). CLE integrated time range ±10 ns.

S6. CLE of h-BN with a 3.65-4.1 eV bandpass filter

The TR photons described in the text show a broad energy spectrum. These are filtered out, if a bandpass is used (Fig S5).

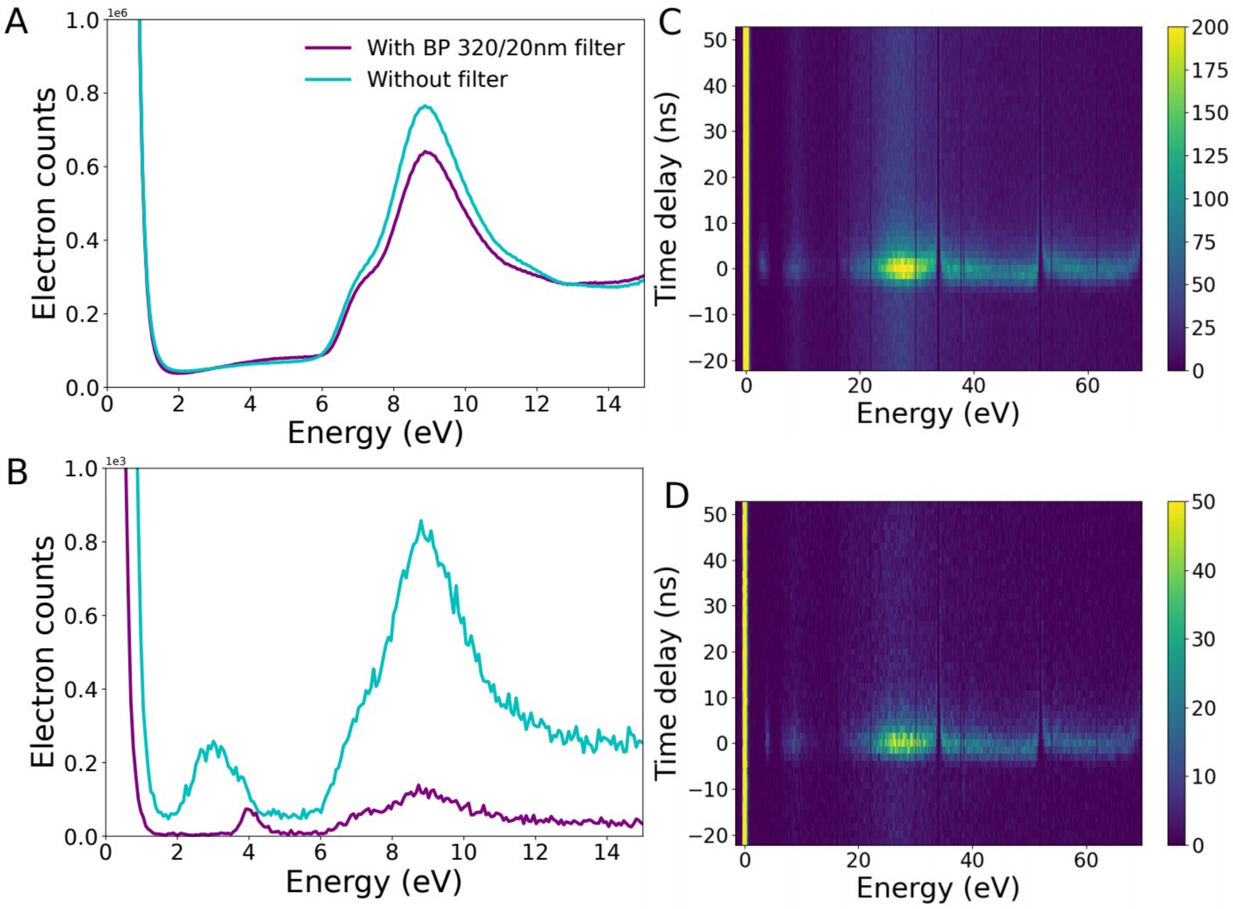

Fig. S5. Effect of filtering the light emission on an h-BN flake: (**A-B**) EELS and CLE of an h-BN flake measured without a filter (PMT response in the 2.0 to 5.0 eV range) and with a bandpass filter (3.65 to 4.1 eV). (**C-D**) 2D histograms for the configurations with and without bandpass filter, respectively.

S7. Spurious coincidence events

First of all, both detectors (a PMT and a Timepix3) used contain noise. For the PMT, these can be remaining photon arriving at the detector from difference sources: ambient light entering the detector, thermal excitation in the photocathode.

Moreover, the electron source used is stochastic, which can lead to spurious correlations. For the current range (10 pA) in our experiments there is 1 electron every 25 ns, on average. Having 2 electrons withing the same detector resolution (our time response function is about 10 ns) would lead to spurious correlations. As a cold FEG is poissonian (for long time delays compared to the emission process) the average number of electron in a 10 ns bin, i.e., during the time between two detection events unresolved by our acquisition chain is: $\lambda = 10/25$ e/ns (the 10 comes from an estimate of our PSF) then the probability to detect within the time bin 0 electrons is: $P(k = 0, \lambda = 0.4) = 0.67$ For one electron the probability is: $P(k = 1, \lambda = 0.4) = 0.25$ And that for more than one electron is: $1 - P(k = 0) – P(k = 1) = 0.08$. This tells us that about 8 % of time bins can contain more than one electron, therefore leading to spurious correlations. That is 0.08/0.033 = 0.24 of all time bins containing one electron or more. To decrease these undesired events and therefore increase the signal to background in CLE, some options are possible. This could be done using a pulsed gun with repetition rate below 1/10 ns^{-1} , using lower emission currents or improving the time response of the detection setup.
